# A Model Report

**Published:** 1884-12

**Authors:** 


A MODEL REPORT.

A model for dental reporters may be found in the account of the papers and discussions of the Dental and Oral Section of the American Medical Association, furnished this journal by the secretary, Dr. John S. Marshall, of Chicago, and concluded in this number. The abstracts of the papers have presented all the salient points without any unnecessary verbiage, and each number has been complete in itself. Rarely has it been the good fortune of any journal to secure such excellent accounts of the doings of any professional body.

